# *ℓ*_0_ Gradient Minimization Based Image Reconstruction for Limited-Angle Computed Tomography

**DOI:** 10.1371/journal.pone.0130793

**Published:** 2015-07-09

**Authors:** Wei Yu, Li Zeng

**Affiliations:** 1 School of Biomedical Engineering, Hubei University of Science and Technology, Xianning, China; 2 College of Mathematics and Statistics, Chongqing University, Chongqing, China; 3 Engineering Research Center of Industrial Computed Tomography Nondestructive Testing of the Education Ministry of China, Chongqing University, Chongqing, China; Zhejiang Univ, CHINA

## Abstract

In medical and industrial applications of computed tomography (CT) imaging, limited by the scanning environment and the risk of excessive X-ray radiation exposure imposed to the patients, reconstructing high quality CT images from limited projection data has become a hot topic. X-ray imaging in limited scanning angular range is an effective imaging modality to reduce the radiation dose to the patients. As the projection data available in this modality are incomplete, limited-angle CT image reconstruction is actually an ill-posed inverse problem. To solve the problem, image reconstructed by conventional filtered back projection (FBP) algorithm frequently results in conspicuous streak artifacts and gradual changed artifacts nearby edges. Image reconstruction based on total variation minimization (TVM) can significantly reduce streak artifacts in few-view CT, but it suffers from the gradual changed artifacts nearby edges in limited-angle CT. To suppress this kind of artifacts, we develop an image reconstruction algorithm based on *ℓ*_0_ gradient minimization for limited-angle CT in this paper. The *ℓ*_0_-norm of the image gradient is taken as the regularization function in the framework of developed reconstruction model. We transformed the optimization problem into a few optimization sub-problems and then, solved these sub-problems in the manner of alternating iteration. Numerical experiments are performed to validate the efficiency and the feasibility of the developed algorithm. From the statistical analysis results of the performance evaluations peak signal-to-noise ratio (PSNR) and normalized root mean square distance (NRMSD), it shows that there are significant statistical differences between different algorithms from different scanning angular ranges (*p*<0.0001). From the experimental results, it also indicates that the developed algorithm outperforms classical reconstruction algorithms in suppressing the streak artifacts and the gradual changed artifacts nearby edges simultaneously.

## Introduction

As an important nondestructive testing method, CT shows large-scale applications in many fields such as medical diagnosis, industrial nondestructive testing, etc. In practical applications of CT imaging, when projection data obtained are adequate and complete, the FBP algorithm, which has been commonly utilized in commercial CT [1], can reconstruct images accurately. However, limited by the scanning environment and the excessive radiation dose imposed to the patients, it is desired that high quality CT images can be reconstructed from low-dose projection data [2,3,4]. To reduce the radiation dose to the patients, an effective imaging modality is X-ray imaging in limited scanning angular range. It is possible that the effective scanning angular range doesn’t satisfy the condition of short scan [5], i.e., the effective scanning angular range is less than 180° plus fan angle. In this case, significant streak artifacts and gradual changed artifacts nearby edges are present in reconstructed images by conventional FBP algorithm and consequently, images are distorted [6]. In the medical domain, especially for dental CT [7,8], C-arm tomosynthesis [9], imaging in the chest and the breast [10] etc., as X-ray ionizing radiation is harmful to human bodies, it is in urgent need to use shorter time of exposure and fewer projection data to reconstruct approximately accurate images. Therefore, to reconstruct high-quality images using limited-angle projection data has been a research focus all along.

Recently, the iterative reconstruction algorithm shows more advantages than conventional FBP algorithm in dealing with the reconstruction problem with incomplete projection data. As early as 1980s, the algebraic reconstruction technique (ART) and simultaneous algebraic reconstruction technique (SART) were utilized by some researchers to investigate CT image reconstruction [11, 12]. While for incomplete projection data, obvious artifacts and noise are present in reconstructed images obtained by the two algorithms.

In 1992, Rudin *et al*. proposed an image denoising method based on total variation (TV) of image [13], and they showed that this method can well protect the edge during the denoising process. Assuming that the pixel value of image at position (*x*,*y*) is labeled by *u*_x,y_, the TV of image can be expressed as

$$||u|{|}_{{}_{TV}}=\sum_{x,y}\sqrt{{\left(u_{x,y}-u_{x-1,y}\right)}^{2}+{\left(u_{x,y}-u_{x,y-1}\right)}^{2}}.$$ (1)

The TV is essentially the *ℓ*_*1*_-norm of the image gradient magnitude. In image domains, images consisting of image gradient magnitudes are approximately sparse. To utilize the sparsity of gradient image, the TV norm can be taken as a regularization function. Furthermore, in 2006, Sidky *et al*. [14] adapted the TV minimization to consider the sparsity of the image gradient magnitudes, and then proposed an accurate algorithm for CT image reconstruction from few-view and limited-angle projection data. This algorithm is called TVM based algorithm hereafter. The TVM based algorithm can obtain accurate images from incomplete projections especially in the sparse angular sampling over 360°. While the scanning angular range is limited and less than 180°(such as 90° and 120°), the reconstruction results suffer from gradual changed artifacts nearby the edges of the objects [14, 15], although it shows superiority to suppress streak artifacts. To further improve the quality of CT images for limited-angle tomography, some scholars have advanced the conventional TV based image reconstruction algorithm [16–18]. Although these methods improve the performance on reducing gradual changed artifacts nearby edges, however, the edge information of the objects may have a certain degree of distortion. With the aim to make the most of previously reconstructed CT images, by means of the constraint of TV, the reconstruction algorithms can generate better images [19,20], while their applications are limited to some extent as the image database is often needed before image reconstruction. Other reconstruction algorithms based on the prior knowledge of image coefficient sparsity in wavelet domains for limited-angle CT image reconstruction can be found in [21–23]. In our work, we focus the regularization in the image domain just as TV regularization done. Thus, the TVM based reconstruction results are compared with our results.

In recent years, a novel regularization method based on the *ℓ*_*0*_-norm of image gradient has been applied in the image smoothing [24], image segmentation [25], image super-resolution and blur deconvolution [26], visual enhancement [27], disparity and optical flow partitioning [28]. Different from the *ℓ*_*0*_-norm of image **u**, ||**u**||_0_, which is the number of its non-zero coefficients, the *ℓ*_*0*_-norm of image gradient is denoted as

$$||\nabla u|{|}_{\;0}=\sum_{p}\#\left\{p|\;|\partial_{x}u_{p}|+|\partial_{y}u_{p}|\neq 0\right\},$$ (2)

where the gradient of 2D image at the pixel point *p* is denoted as ∇*u*_*p*_ = (∂_*x*_ *u*_*p*_,∂_*y*_ *u*_*p*_)^*T*^, ∂_*x*_ *u*_*p*_ and ∂_*y*_ *u*_*p*_ represent the differences in *x* direction and in *y* direction respectively. #{} is counting operator, counting the number of *p* that satisfies |∂_*x*_ *u*_*p*_ *|*+|∂_*y*_ *u*_*p*_ *|≠*0. As the *ℓ*_*0*_-norm of image gradient does not count on gradient magnitude, the large gradient magnitudes will not be penalized, thus the edge can be effectively retained [24].

To better preserve the edges and suppress the artifacts to limited-angle CT image reconstruction, we developed an alternating iterative reconstruction algorithm for limited-angle CT based on *ℓ*_*0*_ gradient minimization. In this paper, different from the *ℓ*_*1*_-norm of the image gradient magnitude mentioned above, the *ℓ*_*0*_-norm of image gradient was taken as the regularization function of the new optimization problem. We converted the optimization problem into a few sub-problems, and solved these problems alternately. From the experimental results, it is shown that by the developed algorithm the streak artifacts and gradual changed artifacts nearby edges can be effectively reduced.

The rest of the paper is organized as follows. In section Method, our reconstruction algorithm for limited-angle tomography is described, together with an efficient numerical scheme. Moreover, the performance evaluations are also outlined in this section. In the following section, experimental results and discussion are presented and conclusions are given in final section.

## Method

**Fig 1:**
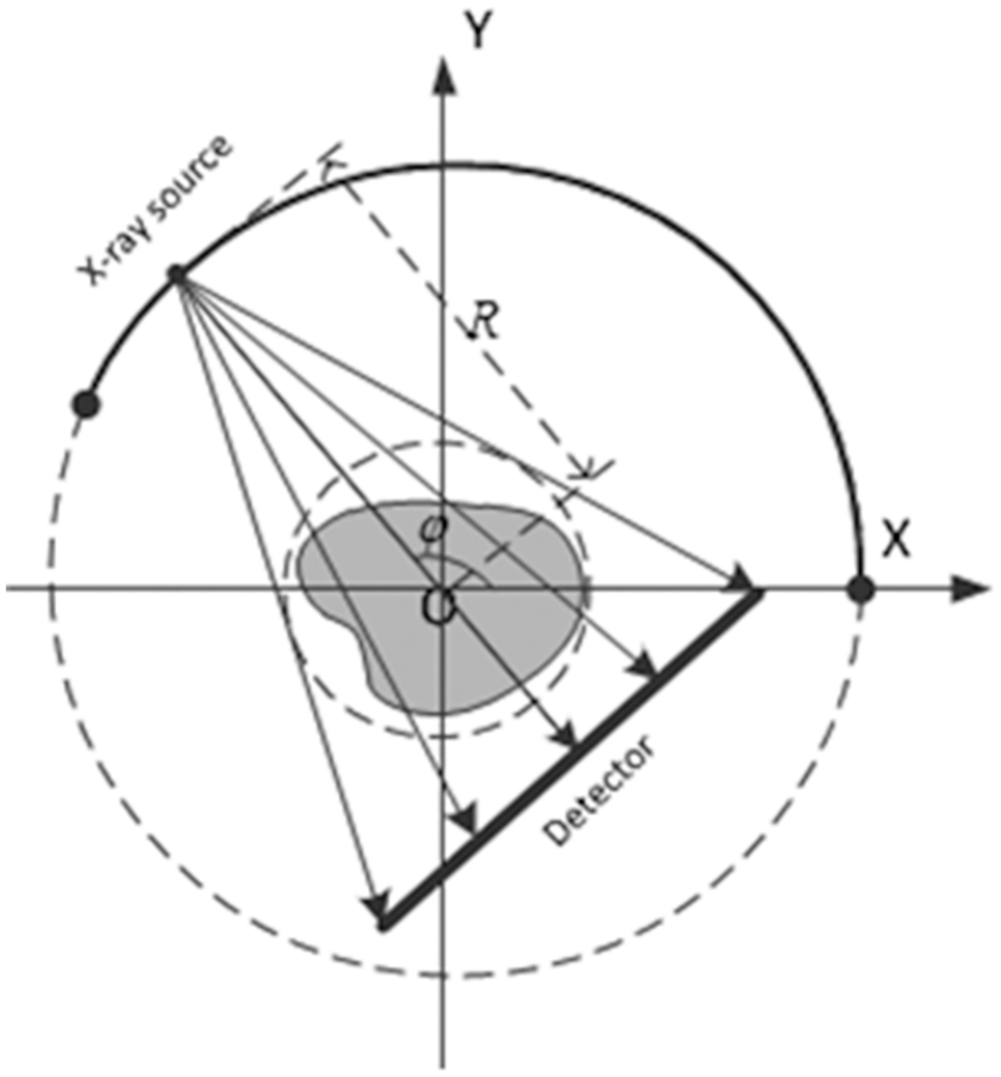
Scanning geometry configuration for circular and limited-angle fan-beam CT.

The fan-beam X-ray CT has been widely used in medical diagnosis, which will be the scanning geometry that we focus in this paper. Fig 1 shows the scanning geometry configuration for circular and limited-angle fan-beam CT. For limited-angle tomography, in this paper, the scanning angular range is limited within [0,*θ*], where *θ* is the maximum rotation angle of the X-ray source, usually less than 180°.

As described in detail in S1 Appendix, we approximate the CT imaging model as following discrete linear system [11]:

Au=g (3)

where **u** is the unknown object to be reconstructed, **g** is the measured projection data, **A** is the system matrix which represents the forward projection.

In some practical CT imaging, when projection data are incomplete, the system Eq (3) is underdetermined. To find the solution to this problem, we usually need to acquire the optimal solution u* satisfying the optimization problem in the following form [29]:

$$u^{*}=\underset{u}{\text{min}}\text{ F(}u\text{):=D(}u\text{)+}\lambda \cdot \text{C(}u\text{).}$$ (4)

where D(**u**) is data fidelity term; C(**u**) represents the regularization term; *λ* is the penalty parameter.

Under the condition that the quality of reconstructed images is ensured, the image reconstruction algorithm based on the regularization constraint is generally employed to further suppress noise and artifacts. In our work, the *ℓ*_*0*_-norm of image gradient served as the regularization constraint term. Then, we developed an image reconstruction model for limited-angle CT:

$$\underset{u}{\text{min}}\text{ F(}u\text{):=}\frac{1}{2}||Au-g|{|}_{2}^{2}\text{+}\lambda \cdot \sum_{p}\#\left\{p||\partial_{x}u_{p}|+|\partial_{y}u_{p}|\neq 0\right\}.$$ (5)

Although the framework of our reconstruction model is similar to general optimization model (4), the regularization term we used is different from other regularization term such as *ℓ*_*1*_-norm of image gradient magnitude, i.e., TV of the image.

In the solution to the optimization problem (5), the original optimization problem was transformed into a few sub-problems which are then calculated in the manner of alternating iteration. The original optimization problem (5) is equivalent to the following sub-problem:

$$u^{n+1}\in \text{arg}\underset{z}{\text{min}}\;{\left(z-u^{n}\right)}^{T}A^{T}\left(Au^{n}-g\right)+\frac{\alpha_{n}}{2}||z-u^{n}|{|}_{2}^{2}+\lambda \cdot \sum_{p}\#\left\{p||\partial_{x}z_{p}|+|\partial_{y}z_{p}|\neq 0\right\}.$$ (6)

Inspired by the work of [29], as demonstrated in Preliminary section of S1 Appendix, the equivalent form of sub-problem (6) is as follows:

$$\left(\text{P1' })\;w^{n}=u^{n}-\frac{1}{\alpha_{n}}\nabla \text{D}(u^{n})=u^{n}-\frac{1}{\alpha_{n}}A^{T}(Au^{n}-g\right)$$ (7)

$$(\text{P2' })\;\underset{z,h,v}{\text{min}}\left\{\sum_{p=1}^{N}{\left(z_{p}-w_{p}^{n}\right)}^{2}+\lambda^{*}\cdot \text{C}(h,v)+\beta ({\left(\partial_{x}z_{p}-h_{p}\right)}^{2}+{\left(\partial_{y}z_{p}-v_{p}\right)}^{2})\right\}$$ (8)

where *α*_n_ and λ* are positive parameter, **A** represents the forward projection, **A** ^***T***^ denoted by the transpose of **A** representing the back projection, $\text{C}(h,v)=\#\left\{p|\;|h_{p}|+|v_{p}|\neq 0\right\}$, *w*_*p*_^*n*^ is the component of **w** ^*n*^ in point *p*, *β* is the regularization parameter that constraints the variables (*h*_*p*_,*v*_*p*_) close to their corresponding gradients (∂_*x*_ *z*_*p*_,∂_*y*_ *z*_*p*_), and the value of *β* is big enough in the experiments.

When solving the sub-problems above, we need to compute for **w** ^*n*^ with **u** ^*n*^ first, then solve the optimization problem (8) with **w** ^*n*^, and then let **u** ^n+1^ = **z** for next iteration. The above alternating minimization algorithm computes an iterative sequence {**u** ^0^,**w** ^1^,**u** ^1^,**w** ^2^,**u** ^2^,**w** ^3^,**u** ^3^,…,**w** ^*n*^,**u** ^*n*^,…}from a given initial value **u** ^0^, which is tailored to the approximation solution of original optimization problem (5).

In Eq (7), the step for computing **w** ^*n*^ is a gradient descent update with a step size of 1/(2α_*n*_) for the problem $w=\underset{u}{\text{argmin}}||Au-g|{|}_{2}^{2}$. To solve the problem, there are many methods, such as conjugate gradient method. As the good property of SART [30], when letting *α*_n_ be the weight for the normalization of the matrix **A** ^*T*^ **A**, Eq (7) becomes SART-type algorithm computing for **w** ^*n*^ as follows:

$$w_{j}^{n}=u_{j}^{n}-\gamma \frac{1}{\overset{M}{\underset{i=1}{\sum }}a_{i,j}}\overset{M}{\underset{i=1}{\sum }}\frac{a_{i,j}}{\overset{N}{\underset{j=1}{\sum }}a_{i,j}}(A_{i}u^{n}-g_{i}),\;j=1,2,\ldots ,N.$$ (9)

where $\sum_{i=1}^{M}a_{i,j}>0$, $\sum_{j=1}^{N}a_{i,j}>0$, **A**_***i***_ is the *i*th row of **A**, γ is weighting factor. **w** ^*n*^ represents the image reconstructed after *n* iterations, each component of **w** ^*n*^ is nonnegative, thus

$$w_{j}^{n}=\left\{ \begin{array}{l} w_{j}^{n}\text{, }w_{j}^{n}\geq 0\\ 0\;,\;w_{j}^{n}<0 \end{array}\right.\text{ , }j=1,2,\ldots ,N.$$ (10)

To solve problem (8), we adopt the alternating minimization algorithm to fix one set of variables while obtain another set of variables and use an accelerated method with the solution in closed form [31]. The detail of how to convert the problem (8) into two sub-problems is listed in S1 Appendix(seen the section of theoretical derivation of our algorithm). From S1 Appendix, it shows that both of the two sub-problems have closed-form solution. Here, we just give the solution of problem (8) as follows:

$$z=F^{-1}\left\{\frac{F(w^{n})+\beta (F^{*}(\partial x)F(h)+F^{*}(\partial y)F(v))}{F(1)+\beta (F^{*}(\partial x)F(\partial x)+F^{*}(\partial y)F(\partial y))}\right\},$$ (11)

Where, for each pixel *p*,

$$\left(h_{p},v_{p}\right)=\left\{ \begin{array}{l} \left(0,0\right)\text{ , }{\left(\partial_{x}u_{p}\right)}^{2}+{\left(\partial_{y}u_{p}\right)}^{2}\leq \frac{\lambda^{*}}{\beta }\\ \left(\partial_{x}u_{p},\partial_{y}u_{p}\right)\;,\text{ otherwise} \end{array}\right..$$ (12)

In summary, the implementation steps of *ℓ*_*0*_-norm gradient based image reconstruction algorithm for limited-angle tomography are given as follows:

**Input:** projection data **g**, max number of reconstruction iterations *N*_*iter*_, initial image **u**_0_,weight λ*, constants *β*_0_ = 2λ*, *β*_max_ = 10^5^ and ratio *κ*.

**Initialization: u** ^**0**^ **= u**_**0**_, *β*←*β*_0_, *n*←0.

**While** stopping criteria is not met **do**

**step 1.** With **u** ^*n*^, compute for **w** ^*n*^ in Eq (9), then non-negative constraint in Eq (10).

**step 2.** *ℓ*_*0*_ gradient minimization

**initialization: z** ^(i)^←**w** ^*n*^, *i*←0.

**repeat**

  with **z** ^*(i)*^, solve for *h*_*p*_^*(i)*^ and *v*_*p*_^*(i)*^ in Eq (12).

  with *h*_*p*_^*(i)*^ and *v*_*p*_^*(i)*^, solve for **z** ^*(i+1)*^ with Eq (11).

*β*←*κβ*, *i*←*i*+1.

**until** *β*≥*β*_max_

**image updating: u** ^n+1^←**z**,

*n*←*n*+1.

**End While**

**Output:** final result **u** ^*n*^

In order to speed up convergence, the parameter *β* is multiplied by *κ* each time starting from a small value *β*_0_, which is automatically adapted in iterations. From the flow chart of Algorithm, we can easily find that the algorithm is implemented in the manner of alternating iteration. In each iteration, there are two steps need to complete. In the first step, the **w** ^*n*^ is computed as SART-type solution in Eq (9). In the second step, we obtain **z** ^*(i+1)*^ with *h*_*p*_^*(i)*^ and *v*_*p*_^*(i)*^ by *ℓ*_*0*_ gradient minimization.

### Performance evaluations

To evaluate the performance of the developed algorithm for limited-angle CT, peak signal-to-noise ratio (PSNR) and normalized root mean square distance (NRMSD) were utilized as follows [32]:

$$PSNR=10{\text{log}}_{10}\left(\frac{MAX^{2}(u_{true})}{\frac{1}{Q}\sum_{m=1}^{Q}{\left(u(m)-u_{true}(m)\right)}^{2}}\right)$$ (13)

$$\text{NRMSD}=\sqrt{\frac{\sum_{m=1}^{Q}{\left(u(m)-u_{true}(m)\right)}^{2}}{\sum_{m=1}^{Q}{\left({\bar{u}}_{true}-u_{true}(m)\right)}^{2}}}$$ (14)

where *u* is the image to be reconstructed, *u*_*true*_ is the phantom image regarded as the original image, the max density value of the original image is denoted as *MAX*(*u*_*true*_) and the average value of the densities of the original image is denoted as ${\bar{u}}_{true}$. *Q* is the total number of pixels of the image. Generally, a higher PSNR indicates that the image is of higher quality. If the image reconstructed is close to the original image, the NRMSD will approach to zero. If there is a large difference in some places, the NRMSD will be large. In addition, if the image reconstructed is uniformly with the correct average density, the NRMSD will be one.

### Statistical Analysis

Statistical analysis is performed on MedCalc statistical software [33]. We test the statistical significance of the performance evaluations PSNR and NRMSD using 20 phases of the NCAT phantom. The F-test is first performed. If the *p*-value of F-test is high (p>0.05), the t-test is performed; If the *p*-value is low (p<0.05), the Welch’s t test [34] is performed. For the statistical significance tests, each variable is expressed as Mean ± standard deviations.

## Results and Discussion

**Fig 2:**
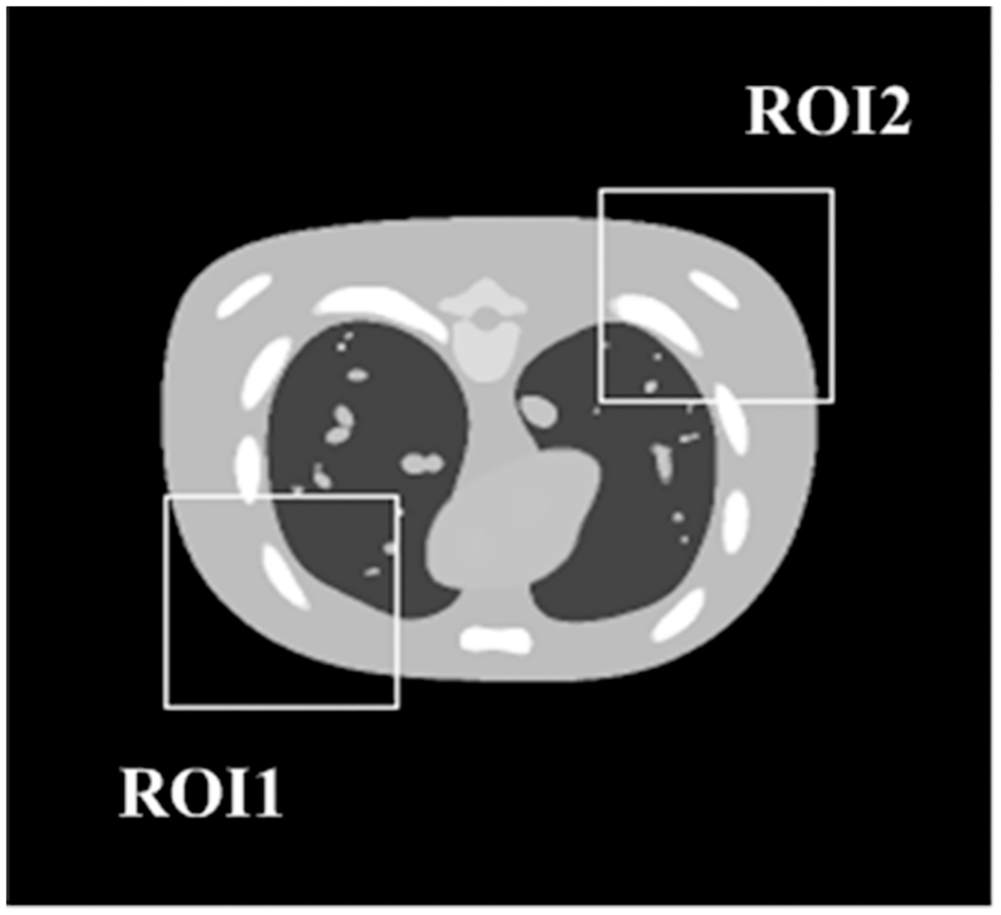
A typical phase of the NCAT phantom.

The experiments are implemented on a 1.8GHz Intel Xeon E5-2603 CPU processor coded in Microsoft Visual C++ 2010. We tested the developed algorithm for limited-angle tomography using a digital NURBS based cardiac-torso (NCAT) phantom with matrix size 256×256 [35–37]. One typical frame of the phantom is shown in Fig 2 (or S1 Fig). In the simulation experiment, assuming the object is fixed, the X-ray source and the detector rotate around the rotation axis synchronously. The simulated geometrical scanning parameters for limited-angle CT are listed in Table 1. The scanning angular ranges investigated are limited in [0,90°] and [0,120°] respectively. Since the sampling interval between two adjacent projection views is 1°, the numbers of the projection views available in above cases are 90 and 120 respectively. For noise-free experiment, the projection data are generated by simulating the forward projection to the discretized NCAT phantom. By adding the Gaussian noise to the noise-free projection data mentioned above, the noisy projection data are generated for noisy experiment. The average value and the standard deviation of the Gaussian noise are zero and 0.1% of the maximum value of the projection data respectively.

**Table 1:** Geometrical scanning parameters for limited-angle CT.

Parameter	Value
Distance between source and detector	1200mm
Distance between source and rotation axis	981mm
Sampling interval between two adjacent projection views	1°
Interval between two adjacent rays per projection view	0.0329°
Number of rays per projection view	256
Diameter of field of view	143.6222mm
Pixel size of the object	0.5632×0.5632mm^2^
Size of reconstruction image	256×256

To validate the developed algorithm, we first compare with other classical iterative reconstruction algorithms for one typical phase of the NCAT phantom: (1) SART algorithm, which has been proved to have more advantage than FBP algorithm when the projection data dose not satisfy the perfect reconstruction condition; (2) TVM based algorithm, which has been widely used for CT image reconstruction from incomplete projection data. In the experiments, we have tested a series of parameters for TVM based algorithm and our algorithm, and choose the parameters with the best image quality for different cases.

In the experiments, the initial image for all iterative algorithms is **u**_0_ = **0**. The weight coefficient *γ* equals to 1.0 in SART-type iteration formula. Reconstruction parameters for TVM based algorithm are used as follows:1) for scanning range [0,90°], *N*_TV_ = 20, *α* = 0.2; 2) for scanning ranges [0,120°], *N*_TV_ = 20, *α* = 0.3. With regard to our algorithm, for scanning ranges [0,90°] and [0,120°], λ* = 0.0001, *κ* = 5. For all the above iterative methods, the stopping criterion is defined as reaching the maximum iteration number *N*_*iter*_ = 1000.

**Fig 3:**
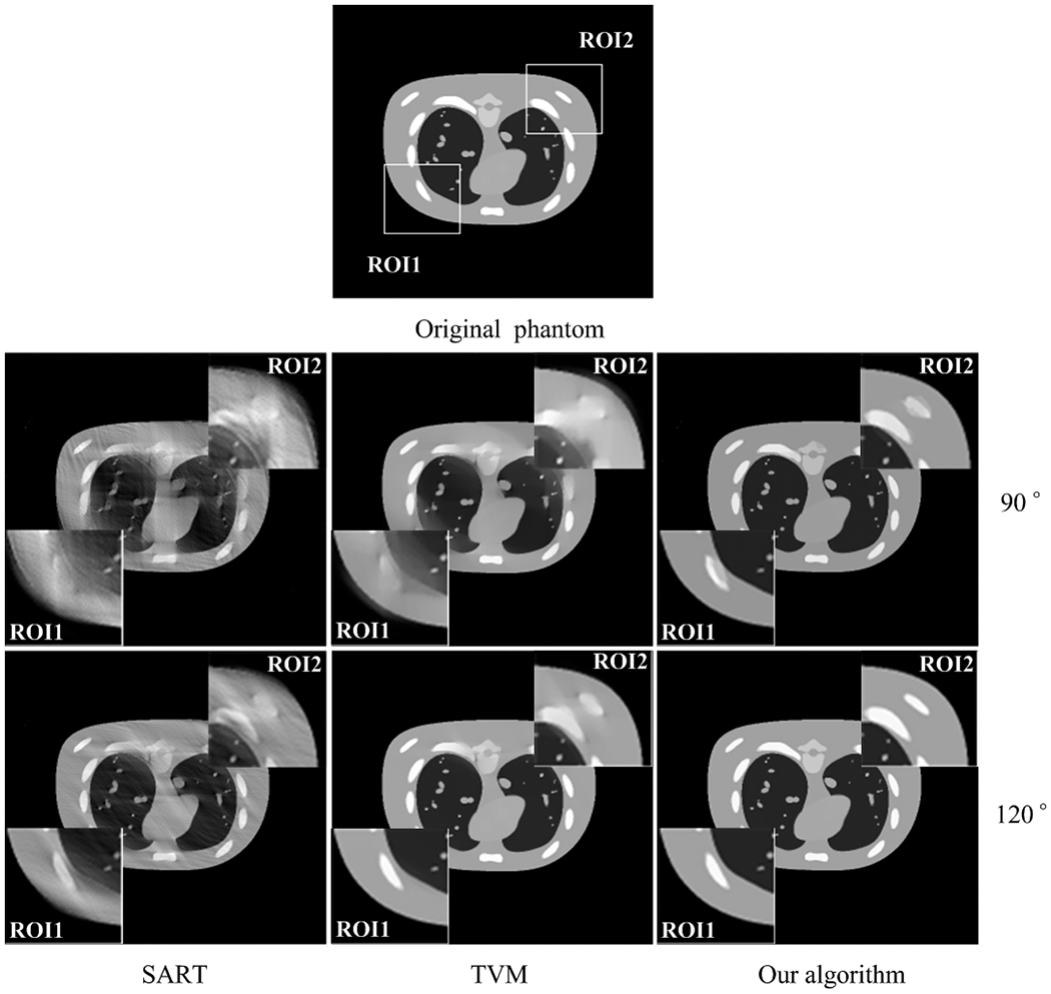
The tomographic results of NCAT phantom from noise-free projection dataset. The image on the top is the original phantom. The following rows are the results reconstructed from scanning angular ranges [0, 90°] and [0, 120°], respectively. Images from left to right in each row present the results reconstructed by SART algorithm, TVM algorithm and our algorithm, respectively. The gray scale window is set to [0, 1.0].

Fig 3 shows the images reconstructed by different algorithms for two different scanning ranges in limited-angle tomography. The image on the top is the original phantom. The following rows are the results reconstructed from scanning ranges [0,90°] and [0,120°], respectively. Images from left to right in each row present the results reconstructed by SART algorithm, TVM based algorithm and our algorithm, respectively. As can be seen from Fig 3, with the increase of the scanning range, the quality of the reconstructed CT images begins to improve with different degrees. Compared to SART algorithm, the streak artifacts can be better suppressed by both the TVM based algorithm and our algorithm. For limited-angle scanning ranges [0,90°] and [0,120°], the gradual changed artifacts nearby edges appear by TVM based algorithm. The reconstructed images are distorted nearby the edges of the object in these cases. However, by our algorithm, the gradual changed artifacts nearby edges can be further reduced and the edge structure information of the object can be better preserved at the mean time.

Table 2 lists the PSNR and NRMSD measures of the images (as shown in Fig 3) reconstructed by different algorithms with 1000 iterations. From Table 2, it finds that our algorithm outperforms the TVM based algorithm and SART algorithm in terms of the PSNR and NRMSD measures. Due to the good property of the regularization function which was defined as the *ℓ*_0_-norm of the image gradient, our algorithm shows better performance than TVM based algorithm. In addition, the experiments show that the larger the scanning ranges, the better the image quality.

**Table 2:** Evaluations of the results reconstructed by different algorithms from noise-free projections shown in Fig 3.

Method	[0, 90°]		[0, 120°]	
	PSNR	NRMSD	PSNR	NRMSD
SART	22.5713	0.2596	24.6687	0.2039
TVM	27.0514	0.1550	37.7364	0.0453
Our algorithm	34.6383	0.0647	40.2834	0.0338

**Fig 4:**
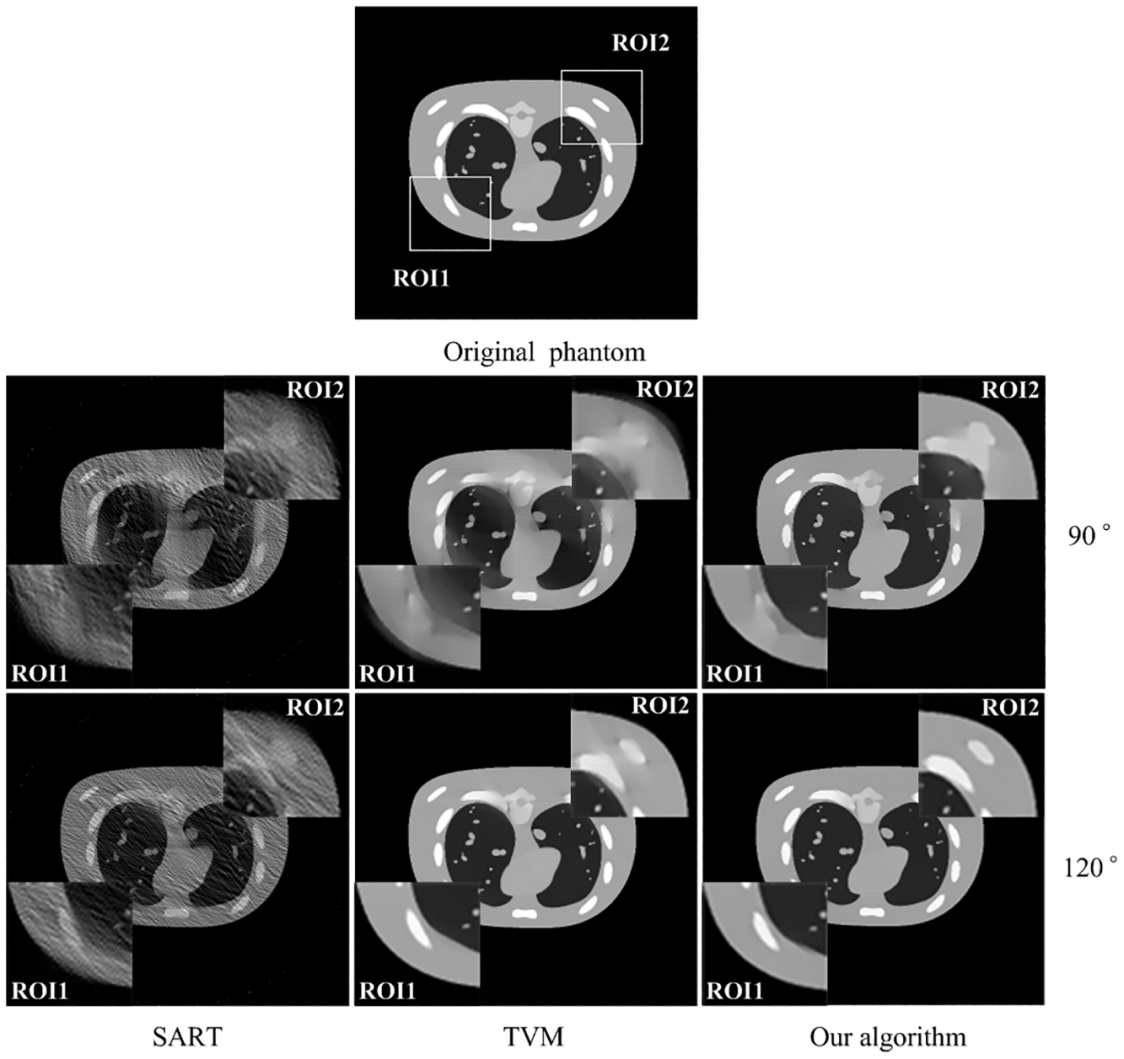
The tomographic results of NCAT phantom from noisy projection dataset. The image on the top is the original phantom. The following rows are the results reconstructed from scanning angular ranges [0, 90°] and [0, 120°], respectively. Images from left to right in each row present the results reconstructed by SART algorithm, TVM algorithm and our algorithm, respectively. The gray scale window is set to [0, 1.0].

In practical applications, the projection data usually contains measurement noise. For the experiments with noisy projection data, reconstruction parameters for TVM based algorithm are used as follows: 1) for scanning range[0,90°], *N*_TV_ = 10, *α* = 0.28; 2) for scanning ranges[0,120°], *N*_TV_ = 20, *α* = 0.3. With regard to our algorithm, the reconstruction parameters used for scanning ranges [0,90°] and [0,120°] are as follows: λ* = 0.0016, *κ* = 7. The stopping criterion for all the above iterative methods is defined as reaching the maximum iteration number 1000. Fig 4 gives the reconstructed images similar to Fig 3 but from a noisy projection dataset. It can be found that the SART algorithm is vulnerable to noise. For scanning ranges[0,90°] and [0,120°], the TVM based algorithm and our algorithm cause fewer artifacts than SART algorithm, while our algorithm recovers even more edge structure information than TVM based algorithm. From Fig 4, it can be seen that by TVM based algorithm, the streak artifacts can be eliminated, while the gradual changed artifacts caused by limited angular scanning still heavily distorted the edge of the objects. And it also illustrates that the reconstructed images by our algorithm have superior visual quality with less gradual changed artifacts nearby edges. Table 3 lists the PSNR and NRMSD measures of the images shown in Fig 4. From Table 3, it shows that the images by our algorithm has better performance than TVM based algorithm and SART algorithm with PSNR and NRMSD.

**Table 3:** Evaluations of the results reconstructed by different algorithms from noisy projections shown in Fig 4.

Method	[0, 90°]		[0, 120°]	
	PSNR	NRMSD	PSNR	NRMSD
SART	18.3931	0.4200	18.3028	0.4244
TVM	25.0899	0.1943	32.2828	0.0849
Our algorithm	27.2007	0.1524	35.9096	0.0559

**Fig 5:**
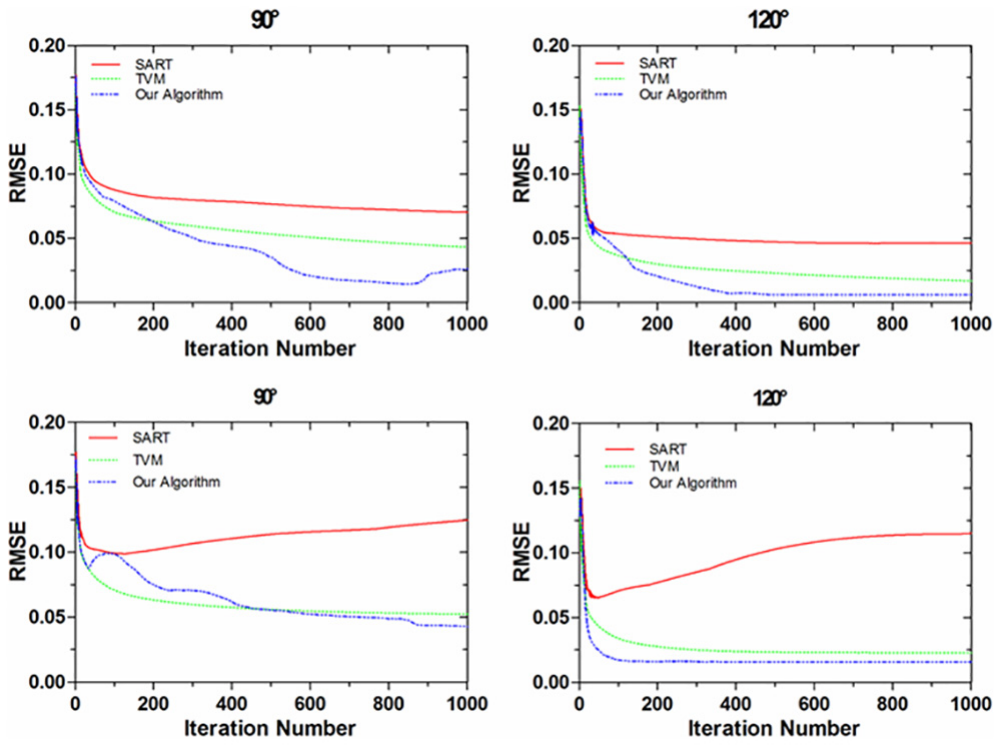
RMSE curves of NCAT phantom reconstructions by SART algorithm, TVM algorithm and our algorithm for one typical phase of the NCAT phantom. The graphs on top row and bottom row present the RMSE curves of the results reconstructed from noise-free projection dataset and noisy projection dataset respectively. The graphs from left to right in each row present the RMSE curves of the results reconstructed from scanning angular ranges [0, 90°] and [0, 120°], respectively.

To further confirm this observation, we have compared root-mean-square error (RMSE) curves of NCAT phantom reconstructions by different algorithms for one typical phase of the NCAT phantom, shown in Fig 5. The graphs on top row and bottom row present the RMSE curves of the results reconstructed from noise-free projection dataset and noisy projection dataset respectively. The graphs from left to right in each row present the RMSE curves of the results reconstructed from scanning ranges [0,90°] and [0,120°], respectively. Our algorithm achieves the minimum of RMSE faster than the other two algorithms. Furthermore, it steadily converges to a low-noisy solution. It demonstrates that we can also get good images when terminating the iteration at earlier stage according to the principle of the minimum of RMSE with small number of iterations. As can be seen from Fig 5, when the scanning range is [0,120°], dozens of iterations are enough for both TVM based algorithm and our algorithm to obtain high quality CT images.

To further assess the performance evaluations of image quality reconstructed by different algorithms, we performed the tests of statistical significance using 20 phases of the NCAT phantom. The statistical analysis results of the images, which are reconstructed from scanning angular range [0,90°] and [0,120°] between different algorithms with 1000 iterations from noise-free projections, are summarized in Tables 4 and 5, respectively. There are significant differences in the values of PSNR and NRMSD between any two algorithms (*p* < 0.0001). From both the Table 4 and Table 5, the values of PSNR by our algorithm are much higher than that of TVM and SART, while the values of NRMSD by our algorithm are much lower than that of TVM and SART. For the experiments that the images reconstructed from noisy projections, the statistical analysis results are summarized in Tables 6 and 7 with different scanning angular ranges. From the Tables 6 and 7, the values of PSNR and NRMSD of our algorithm and TVM based algorithm have significant statistical difference from that of SART (*p* < 0.0001). From Table 6, there are significant statistical differences between our algorithm and TVM based algorithm in PSNR (*p* = 0.0013 < 0.05) and NRMSD (*p* = 0.0036 < 0.05). From Table 7, there are significant statistical differences between our algorithm and TVM based algorithm in PSNR (*p* = 0.0001 < 0.05) and NRMSD (*p* = 0.0002 < 0.05). The values of PSNR and NRMSD shown in the Tables 6 and 7 illustrate that our algorithm has better performance than that of TVM based algorithm and SART algorithm.

**Table 4:** Summary of statistical analysis results of performance evaluations of the images reconstructed from scanning angular range [0, 90°] between different algorithms (with 1000 iterations from noise-free projections for 20 phases of the NCAT phantom).

Item	Method			*p*_*F*_-value			*p*-value		
	SART(A)	TVM(B)	Our algorithm(C)	A vs. B	A vs. C	B vs. C	A vs. B	A vs. C	B vs. C
PSNR	22.8106±0.2356	26.2754±0.6789	34.9005±1.9652	<0.001	<0.001	<0.001	<0.0001	<0.0001	<0.0001
NRMSD	0.2517±0.005809	0.1693±0.01254	0.06409±0.01483	0.002	<0.001	0.472	<0.0001	<0.0001	<0.0001

*p*_*F*_-value: *p*-value of F-test.

*p*-value: If the *p*_*F*_-value is high (*p*>0.05), the t-test is performed; If the *p*_*F*_-value is low (*p*<0.05), the Welch’s t test is performed.

**Table 5:** Summary of statistical analysis results of performance evaluations of the images reconstructed from scanning angular range [0, 120°] between different algorithms (with 1000 iterations from noise-free projections for 20 phases of the NCAT phantom).

Item	Method			*p*_*F*_-value			*p*-value		
	SART(A)	TVM(B)	Our algorithm(C)	A vs. B	A vs. C	B vs. C	A vs. B	A vs. C	B vs. C
PSNR	26.0269±0.5832	35.1035±2.5646	47.8884±4.9335	<0.001	<0.001	0.006	<0.0001	<0.0001	<0.0001
NRMSD	0.1741±0.01207	0.06366±0.01906	0.01682±0.01337	0.054	0.662	0.131	<0.0001	<0.0001	<0.0001

*p*_*F*_-value: *p*-value of F-test.

*p*-value: If the *p*_*F*_-value is high (*p*>0.05), the t-test is performed; If the *p*_*F*_-value is low (*p*<0.05), the Welch’s t test is performed.

**Table 6:** Summary of statistical analysis results of performance evaluations of the images reconstructed from scanning angular range [0, 90°] between different algorithms (with 1000 iterations from noisy projections for 20 phases of the NCAT phantom).

Item	Method			*p*_*F*_-value			*p*-value		
	SART(A)	TVM(B)	Our algorithm(C)	A vs. B	A vs. C	B vs. C	A vs. B	A vs. C	B vs. C
PSNR	18.2747±0.3531	24.7533±0.6350	26.9256±2.5368	0.014	<0.001	<0.001	<0.0001	< 0.0001	0.0013
NRMSD	0.4245±0.01742	0.2016±0.01415	0.1632±0.05083	0.373	<0.001	<0.001	<0.0001	< 0.0001	0.0036

*p*_*F*_-value: *p*-value of F-test.

*p*-value: If the *p*_*F*_-value is high (*p*>0.05), the t-test is performed; If the *p*_*F*_-value is low (*p*<0.05), the Welch’s t test is performed.

**Table 7:** Summary of statistical analysis results of performance evaluations of the images reconstructed from scanning angular range [0, 120°] between different algorithms (with 1000 iterations from noisy projections for 20 phases of the NCAT phantom).

Item	Method			*p*_*F*_-value			*p*-value		
	SART(A)	TVM(B)	Our algorithm(C)	A vs. B	A vs. C	B vs. C	A vs. B	A vs. C	B vs. C
PSNR	18.6296±0.6064	31.1136±1.3193	33.6815±2.2625	0.001	<0.001	0.023	<0.0001	< 0.0001	0.0001
NRMSD	0.4081±0.02933	0.09776±0.01499	0.07438±0.02074	0.005	0.140	0.166	<0.0001	< 0.0001	0.0002

*p*_*F*_-value: *p*-value of F-test.

*p*-value: If the *p*_*F*_-value is high (*p*>0.05), the t-test is performed; If the *p*_*F*_-value is low (*p*<0.05), the Welch’s t test is performed.

**Fig 6:**
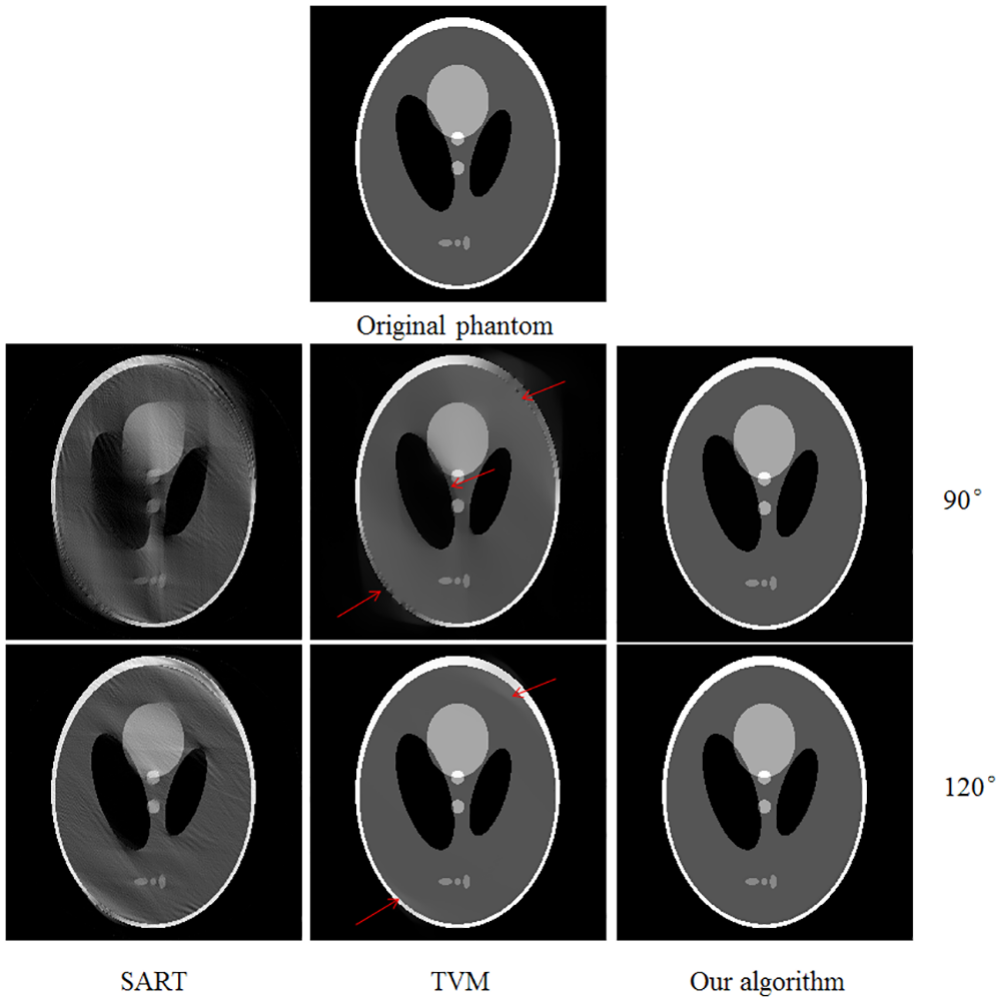
Similar as Fig 3, but the tomographic results of Shepp—Logan phantom.

**Fig 7:**
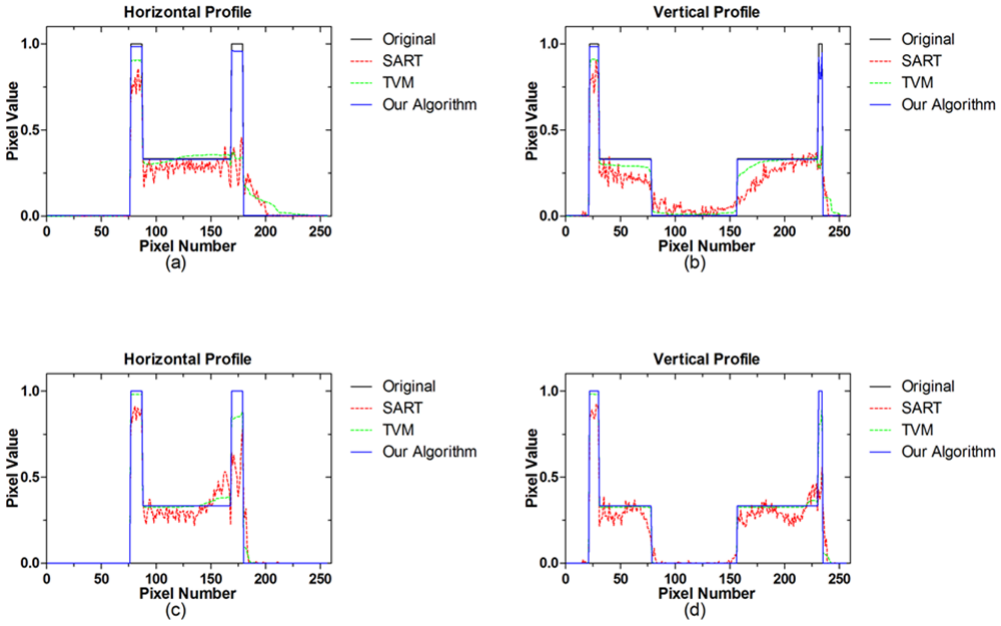
1D profiles of the reconstructed images shown in Fig 6 by different algorithms. The top row and bottom row show the result from scanning angular ranges [0, 90°] and [0, 120°], respectively. (a) and (c) are Horizontal profiles (32th row); (b) and (d) are Vertical profiles(90th column).

In order to further demonstrate the effectiveness of our algorithm, we carry out additional simulation study using the Shepp-Logan phantom. Fig 6 demonstrates the reconstruction results for different scanning angular range by different iterative reconstruction algorithms. From the results demonstrated, it shows that our algorithm has better performance on suppress the gradual changed artifacts nearby edges for limited-angle CT. To further evaluate the performance, the 1D profiles of the images reconstructed by different algorithms are shown in Fig 7. From the profiles, it is indicated that the profiles of our algorithm show better agreement with the original than that of SART and TVM.

## Conclusion

To solve the problem in limited-angle CT image reconstruction, we developed an effective image reconstruction optimization model based on *ℓ*_0_ gradient minimization. The original optimization problem was transformed into a few sub-problems and then, alternating iteration was adopted to calculate this model. In the solution to each sub-problem, features of each sub-problem were fully utilized to generate an effective solution. In this algorithm, the *ℓ*_0_-norm of image gradient was taken as the regularization term to constrain image sparsity. Compared with reconstruction algorithm based on TVM in which the *ℓ*_1_-norm of gradient magnitude acts as the regularization term, our reconstruction algorithm presented in this paper showed more advantages for limited-angle tomography. It was found that from the experiments in limited CT scanning ranges, our reconstruction algorithm caused fewer artifacts in images and could recover edge structure information more effectively. This research investigated the limited-angle image reconstruction problem only in fan-beam CT scanning. In the future, we will investigate the *ℓ*_0_ gradient minimization based CT image reconstruction algorithm for other applications.

## Supporting Information

S1 Fig One typical phase of the NCAT phantom. (TIF)

S1 Appendix Relevant imaging theory and the theoretical derivation of our algorithm. (DOC)
