# Supplementary material for: ℓ 0 Gradient Minimization Based Image Reconstruction for Limited-Angle Computed Tomography
Source: PLoS One. 2015 Jul 9;10(7):e0130793. doi: 10.1371/journal.pone.0130793 (PMC4497654; doi:10.1371/journal.pone.0130793)
Supplement: S1 Appendix — (DOC) [file pone.0130793.s002.doc]

**S1 Appendix.** Relevant imaging theory and the theoretical derivation of our algorithm.

## Imaging model

The fan-beam X-ray CT has been widely used in medical diagnosis, which will be the scanning geometry that we focus in this paper. Fig. 1 shows the scanning geometry configuration for circular and limited-angle fan-beam CT. Let the object center as the origin, in the fixed coordinate system, the X-ray source trajectory can be defined as:

(1)

where $R$ denotes the distance from the X-ray source to the rotational center,  represents the rotating angle of the X-ray source, for circular scanning ,$T$is the transposition operator. For a given rotation angle , the fan-beam projection of the object function is defined as follows [5]:

(2)

where $u(\cdot)$ is the unknown object (X-ray attenuation coefficient) that needs to be reconstructed, is the line integral through the object $u$ in the direction of the unit vector $\mathbf{\alpha}$ from an x-ray source location . The goal of CT image reconstruction is to recover $u$ from the measured projection data . For limited-angle tomography, in this paper, the scanning angular range is limited within $[0,\text{ }\theta]$, where $\theta$ is the maximum rotation angle of the X-ray source, usually less than $180^{\circ}$.

As described in detail previously in [3], we approximate the CT imaging model as a discrete linear system [11]:

$\mathbf{Au=g}.$ (3)

## Preliminary

In mathematics, actually, image reconstruction with limited-angle projection data is taken as an ill-posed inverse problem. To find the solution to this problem, we usually need to acquire the optimal solution $u^{*}$ satisfying the optimization problem in the following form [29]:

$\mathbf{u}^{*}=\min_{\mathbf{u}} \text{ F(}\mathbf{u}\text{):=D(}\mathbf{u}\text{)+}\lambda\cdot\text{C(}\mathbf{u}\text{).}$ (4)

where $\text{D(}\mathbf{u}\text{)}$ is data fidelity term; $\text{C(}\mathbf{u}\text{)}$ represents the regularization term; $\lambda$ is the penalty parameter.

For the general optimization problem (4), Wright *et al.* presented an algorithmic framework and developed separable and approximate iterative optimization method for signal recovery [29]. In their method, the original optimization problem was converted into separable sub-problems. Their approach is suitable for cases in which the sub-problem can be solved much more rapidly than the original problem. Then, for the sub-problem in each step, alternating iteration manner was utilized to acquire the optimal solution$\left\{u^{n},n=0,1,...\right\}$. In this way, the solution obtained was gradually approximate to the real solution to the original optimization problem. The sub-problem to be optimized is demonstrated as follows [29]:

$\mathbf{u}^{n+1}\in\arg\min_{\mathbf{z}} \text{ (}\mathbf{z}-\mathbf{u}^{n}\text{)}^{T}\nabla\text{D}(\mathbf{u}^{n})+\frac{\alpha_{\text{n}}}{2}||\mathbf{z}-\mathbf{u}^{n}||_{2}^{2}+\lambda\cdot\text{C}(\mathbf{z}).$ (5)

where $\alpha_{\text{n}}$ and $\lambda$ are positive parameters; denotes the *ℓ_2_*-norm; $\text{(}\mathbf{z}-\mathbf{u}^{n}\text{)}^{T}$ represents the transpose of the vector $\text{(}\mathbf{z}-\mathbf{u}^{n}\text{)}$; $\mathbf{u}^{n}$ and $\mathbf{u}^{n+1}$ denote the image vectors after $n$ iterations and $n+1$ iterations respectively.

Further analysis indicated that the sub-problem (5) can also be equivalently written as:

$\mathbf{u}^{n+1}\in\arg\min_{\mathbf{z}} \text{ }\frac{1}{2}||\mathbf{z}-\mathbf{w}^{n}||_{2}^{2}+\frac{\lambda}{\alpha_{n}}\cdot\text{C}(\mathbf{z}).$ (6)

where

$\mathbf{w}^{n}=\mathbf{u}^{n}-\frac{1}{\alpha_{n}}\nabla\text{D}(\mathbf{u}^{n}).$ (7)

Specifically, for CT image reconstruction, the data fidelity term is defined by $\text{D(}\mathbf{u}\text{)=}\frac{1}{2}||\mathbf{Au}-\mathbf{g}||_{2}^{2}$. If images are sparse themselves, the regularization term can be expressed as $\text{C(}\mathbf{u}\text{)=}||\mathbf{u}||_{1}$, that is, the *ℓ_1_*-norm of images. Generally, tomographic images themselves are not sparse. Hence, the *ℓ_1_*-norm of image gradient is taken as the regularization term, such as TV [13].

In 2011, Xu *et al.* [24] proposed an image smoothing method using *ℓ_0_*-norm of image gradient as the regularization function which has been applied in many image processing field. Using the *ℓ_0_*-norm of image gradient $||\nabla\mathbf{u}||_{0}$ as the regularization term, that is $\text{C}(\mathbf{u})=\sum_{p} \#\left\{p\left|\text{ }|\partial_{x}u_{p}|+|\partial_{y}u_{p}|\neq0\right.\right\},$ the image smoothing model proposed by Xu *et al.* can be expressed as [24]:

$\min_{\mathbf{u}} \left\{\sum_{p} (u_{p}-v_{p})^{2}+\lambda\cdot(\#\left\{p\left|\text{ }|\partial_{x}u_{p}|+|\partial_{y}u_{p}|\neq0\right.\right\})\right\},$ (8)

where ***v*** is the input image which is known, ***u*** is the output image which is to be estimated. As involving a discrete counting metric in Eq.(8), classical optimization methods (such as gradient decent) are not suited. To make the problem (8) easier to solve, an alternating optimization strategy with half-quadratic splitting was adopted [24]. Although the solution is approximate, the property of maintaining and enhancing salient structures is still upheld[24]. From their work, it is shown that the approximate algorithm is convergent. More details of image smoothing model via *ℓ_0_* gradient minimization can be referenced to the work [24].

## Theoretical derivation of our algorithm

In the paper, the developed image reconstruction model for limited-angle CT is

$\min_{\mathbf{u}} \text{ F(}\mathbf{u}\text{):=}\frac{1}{2}||\mathbf{Au}-\mathbf{g}||_{2}^{2}\text{+}\lambda\cdot\sum_{p} \#\left\{p\left|\text{ |}\partial_{x}u_{p}|+|\partial_{y}u_{p}|\neq0\right.\right\}.$ (9)

In the solution to the optimization problem (9), the original optimization problem was transformed into a few sub-problems which are then calculated in the manner of alternating iteration. The original optimization problem (9) is equivalent to the following sub-problem:

$\mathbf{u}^{n+1}\in\arg\min_{\mathbf{z}} \text{ (}\mathbf{z}-\mathbf{u}^{n}\text{)}^{T}\mathbf{A}^{T}(\mathbf{Au}^{n}-\mathbf{g})+\frac{\alpha_{n}}{2}||\mathbf{z}-\mathbf{u}^{n}||_{2}^{2}+\lambda\cdot\sum_{p} \#\left\{p\left|\text{ |}\partial_{x}z_{p}|+|\partial_{y}z_{p}|\neq0\right.\right\}.$ (10)

Moreover, similarly to (5)-(7), the equivalent form of sub-problem (10) is as follows:

$(\text{P1})\text{ }\mathbf{w}^{n}=\mathbf{u}^{n}-\frac{1}{\alpha_{n}}\nabla\text{D}(\mathbf{u}^{n})=\mathbf{u}^{n}-\frac{1}{\alpha_{n}}\mathbf{A}^{T}(\mathbf{Au}^{n}-\mathbf{g})\text{ }$ (11)

$(\text{P2})\text{ }\mathbf{u}^{n+1}\in\arg\min_{\mathbf{z}} \text{ }\frac{1}{2}||\mathbf{z}-\mathbf{w}^{n}||_{2}^{2}+\frac{\lambda}{\alpha_{n}}\cdot\sum_{p} \#\left\{p\left|\text{ |}\partial_{x}z_{p}|+|\partial_{y}z_{p}|\neq0\right.\right\}$ (12)

In Eq.(12), $\lambda/\alpha_{n}$ is the ratio of two parameters, which can be regarded as an independent parameter, for convenience, the scale parameter $\lambda/\alpha_{n}$ is labeled with $\lambda^{*}$. Then, an equivalent form of sub-problem (12) is

$\mathbf{u}^{n+1}=\arg\min_{\mathbf{z}} \text{ }||\mathbf{z}-\mathbf{w}^{n}||_{2}^{2}+\lambda^{*}\cdot\sum_{p} \#\left\{p\left|\text{ |}\partial_{x}z_{p}|+|\partial_{y}z_{p}|\neq0\right.\right\}.$ (13)

It is well known that the original *ℓ_0_*-norm regularized optimization problem is difficult to solve. In order to solve for $\mathbf{z}$ in Eq.(13), we adopt an approximate method [24], which make the problem easier to tackle, by introducing another two auxiliary variables $h_{p}$ and $v_{p}$ corresponding to the gradient $\partial_{x}z_{p}$ in x-direction and the gradient $\partial_{y}z_{p}$ in y-direction. Then, we have following sub-problems

$(\text{P1' })\text{ }\mathbf{w}^{n}=\mathbf{u}^{n}-\frac{1}{\alpha_{n}}\nabla\text{D}(\mathbf{u}^{n})=\mathbf{u}^{n}-\frac{1}{\alpha_{n}}\mathbf{A}^{T}(\mathbf{Au}^{n}-\mathbf{g})$ (14)

$(\text{P2' })\text{ }\min_{\mathbf{z},\mathbf{h},\mathbf{v}} \left\{\sum_{p=1}^{N} (z_{p}-w_{p}^{n})^{2}+\lambda^{*}\cdot\text{C}(\mathbf{h},\mathbf{v})+\beta((\partial_{x}z_{p}-h_{p})^{2}+(\partial_{y}z_{p}-v_{p})^{2})\right\}$ (15)

where $\alpha_{n}$ and $\lambda^{*}$ are positive parameter, $\mathbf{A}$represents the forward projection, $\mathbf{A}^{T}$denoted by the transpose of $\mathbf{A}$representing the back projection, $\text{C}(\mathbf{h},\mathbf{v})=\#\left\{p\left|\text{ }|h_{p}|+|v_{p}|\neq0\right.\right\}$, $w_{p}^{n}$ is the component of $\mathbf{w}^{n}$ in point $p$,$\beta$ is the regularization parameter constrainting the variables $(h_{p},v_{p})$ similar to their corresponding gradients $(\partial_{x}z_{p},\partial_{y}z_{p})$. Eq.(15) is equivalent to Eq.(13) when $\beta\to\infty$.

For Eq.(14), the main body of the paper has analysed and using SART-type algorithm to compute for $\mathbf{w}^{n}$.

To solve problem (15), we adopt the alternating minimization algorithm to fix one set of variables while obtain another set of variables. Then, the problem (15) can be decomposed into the following two sub-problems.

1. **Sub-problem A of problem (15)**: computing $(\mathbf{h},\mathbf{v})$

We fix $\mathbf{z}$, then the corresponding optimization sub-problem is:

$\min_{\mathbf{h},\mathbf{v}} \left\{\sum_{p=1}^{N} ((\partial_{x}z_{p}-h_{p})^{2}+(\partial_{y}z_{p}-v_{p})^{2})+\frac{\lambda^{*}}{\beta}\text{C}(\mathbf{h},\mathbf{v})\right\}.$ (16)

where the *ℓ_0_*-norm of gradient can be modeled as:

$\text{C}(\mathbf{h},\mathbf{v})=\sum_{p} H(\left|h_{p}\right|+\left|v_{p}\right|).$ (17)

and

$H(\left|h_{p}\right|+\left|v_{p}\right|)=\left\{\begin{matrix} 1,\text{ }\left|h_{p}\right|+\left|v_{p}\right|\neq0\text{ } \\ 0,\text{ otherwise} \end{matrix}\right..$ (18)

Therefore, Eq.(16) can be further decomposed to

$\sum_{p=1}^{N} \min_{h_{p},v_{p}} \left\{(\partial_{x}u_{p}-h_{p})^{2}+(\partial_{y}u_{p}-v_{p})^{2}+\frac{\lambda^{*}}{\beta}H(\left|h_{p}\right|+\left|v_{p}\right|)\right\}.$ (19)

If we can obtain the minimum of Eq.(19) for each pixel $p$, summing all of them, i.e., $\sum_{p} E_{p}^{*}$, can get the global optimal value for Eq.(19). For this purpose, we consider solving the minimum of energy function $E_{p}$ for each pixel $p$ in Eq.(19) as follows:

$E_{p}=\left\{(\partial_{x}u_{p}-h_{p})^{2}+(\partial_{y}u_{p}-v_{p})^{2}+\frac{\lambda^{*}}{\beta}H(\left|h_{p}\right|+\left|v_{p}\right|)\right\}.$ (20)

Solving $(h_{p},v_{p})$ is equivalent to determine the condition that the energy function $E_{p}$ reaches its minimum $E_{p}^{*}$. As for Eq.(20), it is easy to obtain the optimal condition:

$(h_{p},v_{p})=\left\{\begin{matrix} (0,0)\text{ , }(\partial_{x}u_{p})^{2}+(\partial_{y}u_{p})^{2}\leq\frac{\lambda^{*}}{\beta} \\ (\partial_{x}u_{p},\partial_{y}u_{p})\text{ },\text{ otherwise} \end{matrix}\right..$ (23)

1. **Sub-problem B of problem (15)**: computing $\mathbf{z}$

We fix $(\mathbf{h},\mathbf{v})$, then the corresponding optimization sub-problem is:

$\min_{z} \left\{\sum_{p=1}^{N} (z_{p}-w_{p}^{n})^{2}+\beta((\partial_{x}z_{p}-h_{p})^{2}+(\partial_{y}z_{p}-v_{p})^{2})\right\}.$ (24)

The objective function here is a quadratic function which has a global optimal solution even by conventional gradient decent method. In this paper, to solve (22) we use an alternative method which is considered to be a faster method. By using the convolution theorem of Fourier Transform and the diagonalized derivative operator, Wang *et al.* provided an alternating minimization algorithm for total variation image reconstruction with the solution in closed form [31], which can accelerate the speed of convergence. By that method, we can obtain the solution in closed form of our problem (22) and use the Fast Fourier Transform (FFT) to accelerate the solving process of (22).

$z=F^{-1}\left\{\frac{F(w^{n})+\beta(F^{*}(\partial x)F(h)+F^{*}(\partial y)F(v))}{F(1)+\beta(F^{*}(\partial x)F(\partial x)+F^{*}(\partial y)F(\partial y))}\right\}.$ (23)

where $F$ and $F^{*}()$ denote the Fast Fourier Transform operator and its the complex conjugate respectively. The operators here (such as addition, multiplication and division) are all component-wise.
